# Mitral Valve Transcatheter Edge-to-Edge Repair Volumes and Trends

**DOI:** 10.1155/2023/6617035

**Published:** 2023-12-19

**Authors:** Kris Kumar, Timothy F. Simpson, Harsh Golwala, Adnan K. Chhatriwalla, Scott M. Chadderdon, Robert L. Smith, Howard K. Song, Ryan R. Reeves, Paul Sorajja, Firas E. Zahr

**Affiliations:** ^1^Knight Cardiovascular Institute, Oregon Health and Science University, Portland, OR, USA; ^2^Sulpizio Cardiovascular Center, University of California San Diego, La Jolla, CA, USA; ^3^Saint Luke's Mid America Heart Institute, University of Missouri-Kansas City, Kansas City, MO, USA; ^4^The Heart Hospital, Baylor Scott and White, Plano, TX, USA; ^5^Minneapolis Heart Institute, Abbott Northwestern Hospital, Minneapolis, MN, USA

## Abstract

**Background:**

Despite an association between operator volumes and procedural success, there remains an incomplete understanding of the contemporary utilization and procedural volumes for mitral valve transcatheter edge-to-edge repair (MTEER). We aimed to identify annual operator procedural volumes, temporal trends, and geographic variability for MTEER among Medicare patients in the United States (US).

**Methods:**

We queried the National Medicare Provider Utilization and Payment Database for a CPT code (33418) specific for MitraClip device from 2015 through 2019. We analyzed annual operator procedural volumes and incidence and identified longitudinal and geographic trends in MTEER utilization.

**Results:**

From 2015 through 2019, a total of 27,034 MTEER procedures were performed among Medicare patients in the US. The nationwide incidence increased from 6.2 per 100,000 patients in 2015 to 23.8 per 100,000 patients in 2019, a 283% increase over the study period (*P*_trend_ < 0.001). The incidence of MTEER by state varied by nearly 900% (range 5.5 to 54.9 per 100,000 person-years). In 2019, the mean annual MTEER operator annual volume was 9.1 MTEER procedures and had grown from 6.2 per year in 2015.

**Conclusions:**

In this nationwide study of Medicare beneficiaries in the United States, we identified a significant and sustained increase in the utilization of MTEER devices and operators and growth in annual procedural volumes from 2015 through 2019 with considerable variability in utilization by state. Further studies are needed to understand the clinical impact of variability in utilization and the optimal procedural volumes to ensure high efficacy outcomes and maintain critical access to MTEER therapies.

## 1. Introduction

Severe mitral regurgitation (MR) is associated with poor prognosis and survival [1–7]. Treatment of MR via surgical repair or replacement is complex due to anatomic and geometric features of the valvular apparatus and the left ventricle and can carry a high surgical risk as a result of comorbid conditions within the population [8–10]. The randomized EVEREST-II trial evaluated the use of mitral valve transcatheter edge-to-edge repair (MTEER) via the MitraClip system (Abbott Vascular, Chicago IL) versus surgical repair or replacement and showed similar clinical and better safety outcomes in patients with high surgical risk and degenerative MR [11]. Based on these findings and results from the EVEREST-II High Risk Registry [12], the Food and Drug Administration (FDA) granted approval of the MitraClip device for degenerative MR on October 24^th^, 2013. Following this, the randomized controlled COAPT trial demonstrated reduced hospitalization and cardiac death in functional MR patients with MTEER as compared to optimal medical therapy alone [13], resulting in FDA approval of MitraClip system for functional MR on March 14^th^, 2019.

Approval of MitraClip has led to further development of structural heart programs beyond transcatheter aortic valve replacement (TAVR) and an expansion of operator familiarity in performing these procedures in patients with higher surgical risk. As a stipulation of coverage, the US Centers for Medicare and Medicaid Services (CMS) implemented minimal volume requirements for institutions (≥20 MV procedures of which at least 10 are repairs) and operators for both surgical valve procedures (≥20 MV surgeries of which at least 50% are repairs) and structural heart interventions (≥50 structural interventions or ≥ 30 left-sided procedures) as a surrogate to establish quality outcomes. Despite an association with operator volumes and procedural success and rates of complications from coronary intervention datasets [14–16], there remains an incomplete understanding of the contemporary operator procedural volumes and trends in the utilization of MTEER devices. Thus, we aimed to evaluate the current utilization, trends in operator volumes, and geographic incidence of MTEER using the MitraClip edge-to-edge device in the United States for Medicare beneficiaries using the National Medicare Provider Utilization and Payment Database (MPUPD).

## 2. Methods

We assessed MTEER procedural volumes, trends, and geographic distribution across the United States from the year 2015 to 2019 by utilizing MPUPD. The MPUD is a free, publicly available administrative claims database available online [17] by the United States Center for Medicare and Medicaid Services which includes all services for patients within the fee for service Medicare and Medicaid population. The MPUD was analyzed from calendar years 2015 to 2019 (calendar year 2019 is the latest complete dataset available at the time of manuscript preparation) for Current Procedural Terminology (CPT) code 33418. While volumes are included in summary data, MTEER operators performing fewer than 10 procedures per year are excluded from MPUPD for privacy concerns (name of operators, National Provider Identifier numbers, and operator volume <10 procedures per year).

This study was exempt from the Institutional Review Board oversight as we utilized publicly available database files in which patient level data are deidentified.

### 2.1. Statistical Analysis

We identified MTEER volumes as assessed via billed procedures through institution reported CPT coding from 2015 to 2019. A change of volume over time is reported as percent change from the initial year of reporting to the last year of reporting (2015–2019) and year-to-year changes. Operator volumes (either primary or secondary operator) were assessed based upon physician submitted claims as per MTEER CPT codes. Descriptive statistics were utilized to assess operator volumes and compared using analysis of variance modeling. Geographic distribution of MTEER operators and procedural volumes were assessed based upon the United States Census Bureau boundaries for state lines and population data.

## 3. Results

**Figure 1:**
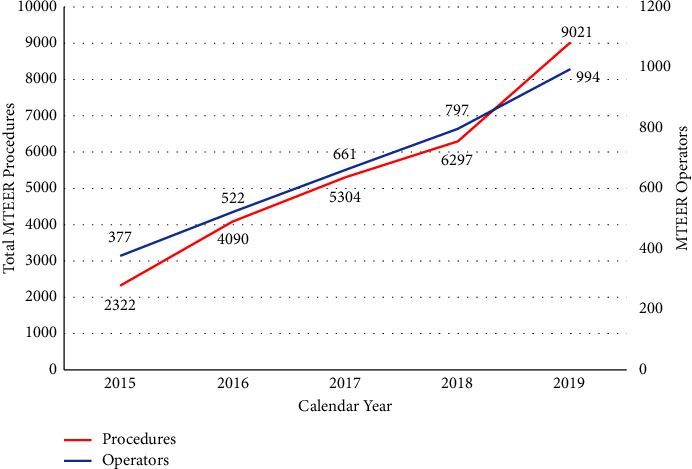
Total mitral valve transcatheter edge-to-edge (MTEER) procedure and operator volumes over the study period from calendar years 2015 to 2019.

**Figure 2:**
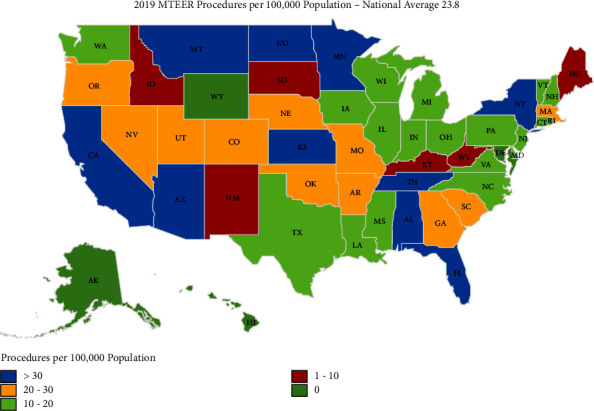
Geographic distribution within the United States of mitral valve transcatheter edge-to-edge (MTEER) procedures per 100,000 population in 2019.

During the study period of 2015 to 2019, a total of 27,034 MTEER procedures were performed among Medicare beneficiaries in the United States. Procedural volume increased from 2322 to 9021 cases during the study period, representing a 283% increase in MTEER procedures during the study period (*P*_trend_ < 0.001) (Figure 1). There was an increase in MTEER procedures from 6.2 per 100,000 patients in 2015 to 23.8 per 100,000 patients in 2019 (Figure 2). The number of operators performing MTEER procedures increased from 377 to 994 during the study period, corresponding to an increase in operators from 1.01 to 2.62 per 100,000 Medicare patients.  Table 1 lists the total procedural volume, total MTEER operators, mean MTEER operator volume, and operator density per 100,000 among Medicare beneficiaries in the calendar year 2019.

**Figure 3:**
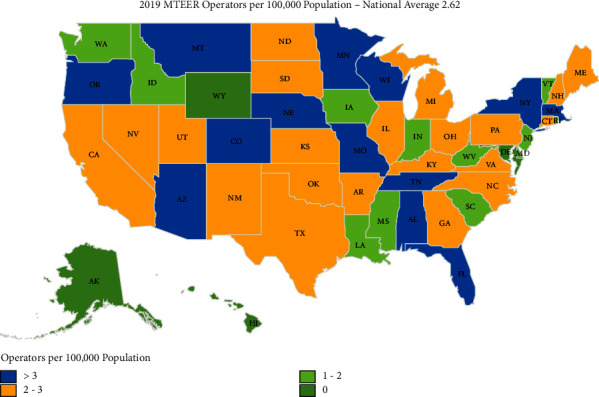
Geographic distribution within the United States of mitral valve transcatheter edge-to-edge (MTEER) operators per 100,000 population in 2019.

**Figure 4:**
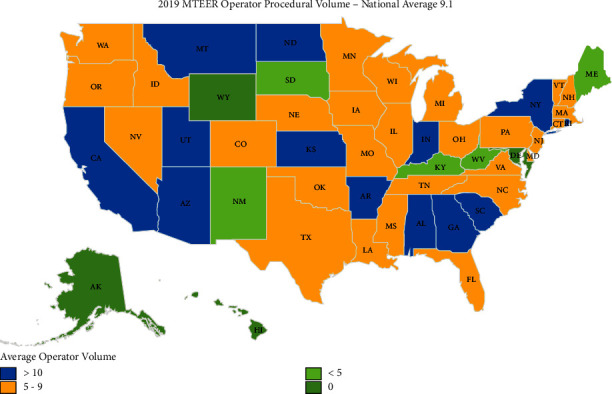
Geographic distribution within the United States of mitral valve transcatheter edge-to-edge (MTEER) operator average procedural volume in 2019.

By state, procedural utilization of MTEER in 2019 varied from 5.5 per 100,000 Medicare beneficiaries in Maine to 54.9 per 100,000 in Montana (900% variation). Four states reported 10 or fewer MTEER procedures to Medicare beneficiaries during the study period, Alaska, Delaware, Hawaii, and Wyoming.  Figure 3 demonstrates the geographic distribution of the number of MTEER operators per 100,000 in the year 2019 while Figure 4 demonstrates the geographic distribution of the mean MTEER procedures performed per operator in 2019.

**Figure 5:**
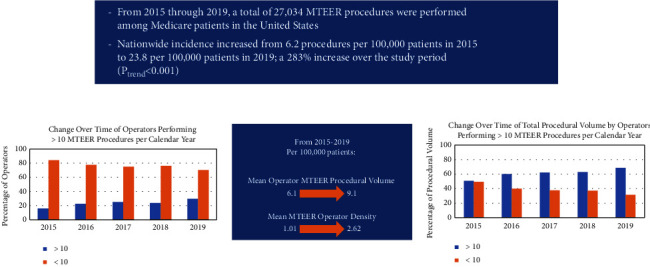
Changes in mitral valve transcatheter edge-to-edge (MTEER) overall procedure and operator volumes, as well as distribution of volumes for operators performing >10 procedures per year.

Operators performing greater than 10 procedures per year increased over time, from 16% of operators in 2015 to 30% of operators in 2019 (Figure 5). The percentage of total procedures performed by operators performing greater than 10 procedures per year increased from 51% of total procedures in 2015 (1176 procedures) to 68% of total procedures in 2019 (6176 procedures).

The top 1% of operators in 2019 performed 7.7% of all MTEER procedures for the calendar year 2019. From 2015 to 2019, the top 1% of operators demonstrated an increase in procedural volume by a mean of 318% during the study period. From 2015 to 2019, the number of operators performing cumulative procedures greater than 200 was 5 (0.5%), greater than 100 was 34 (3.4%), and greater than 50 was 77 (7.7%).

## 4. Discussion

In our analysis of Medicare patients in the United States, we identified a significant increase in the incidence of MTEER utilization, total number of MTEER operators, and mean annual operator volume during the study period. In addition, we identified geographic disparities in utilization by state.

Following FDA approval for the MitraClip device, there has been a significant rise nationally in MTEER procedural volumes nationally. As the elderly population within the United States continues to grow, there is a higher prevalence of valvular disease which increases with age [18]. MR affects more than 2 million individuals nationally, with rates of mortality for severe MR up to 6% per year [19]. As MR is a significant cause of mortality and heart failure hospitalization, patients are being increasingly referred for possible MTEER to improve outcomes in this population. In our analysis, we demonstrate sustained growth of both operators and procedural volumes nationally during the study period. In addition, following the publication of the COAPT trial results and subsequent FDA approval for functional MR, there was an increase in the rate of rise of MTEER procedures compared to previous years with sustained and steady growth of MTEER operators.

As our study analyzed the MPUD database through the calendar years 2015 to 2019, the FDA approval for both degenerative and functional MR (October 24^th^, 2013, and March 14^th^, 2019, respectively) was included in this analysis; however, CMS approval for both indications was not fully reflected. Degenerative MR CMS approval occurred on August 7^th^, 2014, and volumes as a result of CMS approval for this indication are reflected. Conversely, functional MR achieved CMS approval on January 29^th^, 2021, and as a result, volumes are not reflected in this analysis due to MPUD data only being available through the 2019 calendar year. We anticipate as further datasets become available and are reflective of CMS approval for both indications, volumes will continue to rise for MTEER based upon CPT code analysis.

Nationally, the rise of structural heart disease training programs and centers performing transcatheter therapies with heart team approaches beginning with TAVR has led to an expansion of the field [20, 21] leading to centers performing MTEER and transcatheter mitral valve replacement as well as dedicated transcatheter tricuspid valve therapies. While the mean TAVR volume per operator essentially plateaued to 23 TAVR procedures per operator during the years 2017 and 2018 [22], procedural volumes and operators for MTEER continue to show sustained growth as demonstrated in our analysis. However, the total number of MTEER operators still is significantly lower than those performing TAVR. This is likely reflective of several factors including patient risk profiles for degenerative MR, more recent FDA approval of the device, operator familiarity and training with the complexity and challenging nature of the MTEER procedure, and necessity of dedicated structural trained cardiologists specializing in multimodality imaging for both pre- and periprocedural guidance. In addition, as MTEER is relatively newer compared to TAVR with regards to CMS approval, access to therapies is largely within Level 1 centers which specialize in these transcatheter-based therapies for mitral and tricuspid valve interventions.

Improved outcomes and optimal procedural success of MTEER are closely linked to operator experience, with decreases in complications and procedural times as operator experience increases with an operator learning curve that may approach 200 cumulative cases [23]. This study identifies with the highest absolute and relative procedure volume, highlighting areas of the country where gaps may exist in the availability of care. The number of operators performing greater than 10 procedures per year has steadily increased since 2015, though has remained at 30% of operators as of 2019. Of these operators, they have performed the majority of procedures, rising from 51% of total procedures by experienced operators in 2015 to 68% of total procedures in 2019. MTEER is a procedure that is both challenging in terms of technical complexity as well as the understanding of advanced echo imaging for successful MTEER deployment and procedural success. Thus, there is a rationale for MTEER program building with higher volume operators initially performing procedures first and then proctoring to train newer operators in MTEER.

Improving access to these therapies in states where MTEER volumes are low and a lower density of MTEER exists can help improve access to procedures to patients of a wider range of geographic, socioeconomic, and racial and ethnic backgrounds [24, 25]. While overall volume has been linked to procedural success, currently MTEER compared to TAVR is a relatively new field, and transcatheter device technology continues to evolve which can further improve clinical outcomes.

### 4.1. Limitations

Our study has a few important limitations to be addressed. First, our analysis of the MPUD database is limited to Medicare beneficiaries; thus, patients outside of the Medicare age group and private insurers were not included which contributes to the total number of procedures performed throughout the United States. However, younger patients often at a lower surgical risk are more likely to be surgical candidates, and the Medicare population via the MPUD database is likely a reflection of more real-world use of MTEER devices in a higher risk cohort. In addition, an inherent limitation of the MPUD database exists with the potential for incomplete datasets during time periods where new programs were developed during a particular year as well no clinical or procedural outcomes linked to the database. Lastly, the geographic limitations of stratifying data by state do not take into account populations and urban centers that may span across state lines.

## 5. Conclusions

In conclusion, our analysis of the MPUD database reveals an increase in the amount of MTEER procedures and operators both overall and per 100,000 population within the United States from 2015 to 2019. There is significant geographic variation in the distribution of operators performing these procedures, with a majority of the total procedures during the study period performed by operators performing greater than 10 procedures per year.

## Figures and Tables

**Table 1:** Mitral valve edge-to-edge repair (MTEER) volumes, operators, and operator density within the United States among Medicare beneficiaries during the calendar year 2019.

	MTEER volume	MTEER operators	Average MTEER volume per operator	Operator density per 100,000 population
United States	**9,021**	**994**	**9.1**	**2.62**
Alabama	284	24	11.8	3.96
Alaska	0	0	—	—
Arizona	333	24	13.9	3.04
Arkansas	135	10	13.5	2.13
California	1,127	83	13.6	2.35
Colorado	145	20	7.3	3.77
Connecticut	78	11	7.1	2.76
Delaware	0	0	—	—
District of Columbia	24	5	4.8	6.61
Florida	936	101	9.3	4.11
Georgia	300	29	10.3	2.79
Hawaii	0	0	—	—
Idaho	20	4	5.0	1.82
Illinois	257	38	6.8	2.34
Indiana	103	9	11.4	1.05
Iowa	66	8	8.3	1.65
Kansas	234	9	26.0	2.07
Kentucky	51	15	3.4	2.45
Louisiana	72	10	7.2	1.87
Maine	12	6	2.0	2.76
Maryland	113	13	8.7	1.43
Massachusetts	250	31	8.1	3.11
Michigan	183	29	6.3	2.42
Minnesota	266	32	8.3	5.76
Mississippi	76	9	8.4	1.88
Missouri	189	26	7.3	3.37
Montana	102	7	14.6	3.77
Nebraska	73	10	7.3	3.47
Nevada	69	7	9.9	2.11
New Hampshire	29	5	5.8	2.06
New Jersey	141	22	6.4	1.94
New Mexico	20	7	2.9	2.61
New York	709	65	10.9	3.07
North Carolina	218	26	8.4	2.09
North Dakota	38	3	12.7	2.81
Ohio	247	33	7.5	2.49
Oklahoma	121	13	9.3	2.25
Oregon	111	16	6.9	3.40
Pennsylvania	277	47	5.9	3.00
Rhode Island	23	2	11.5	1.67
South Carolina	153	12	12.8	1.59
South Dakota	11	3	3.7	2.17
Tennessee	294	30	9.8	3.69
Texas	445	53	8.4	2.13
Utah	71	7	10.1	2.79
Vermont	14	2	7.0	1.55
Virginia	273	28	9.8	2.38
Washington	159	16	9.9	1.78
West Virginia	17	5	3.4	1.73
Wisconsin	125	21	6.0	3.17
Wyoming	0	0	—	—

MTEER = mitral valve transcatheter edge-to-edge repair.

## Data Availability

The data used to support the study are publicly available via the National Medicare Provider Utilization Database (available from https://www.cms.gov/Research-Statistics-Data-and-Systems/Statistics-Trends-and-Reports/Medicare-Provider-Charge-Data/Physician-and-Other-Supplier).

## Abbreviations

CMS: Centers for Medicare and Medicaid
MPUD: National Medicare Provider Utilization Database
MR: Mitral regurgitation
MTEER: Mitral valve transcatheter edge-to-edge repair
MV: Mitral valve
STS: Society of Thoracic Surgeons.

## References

[1] Antoine C., Benfari G., Michelena H. I. (2018). Clinical outcome of degenerative mitral regurgitation: critical importance of echocardiographic quantitative assessment in routine practice. *Circulation*.

[2] Asgar A. W., Mack M. J., Stone G. W. (2015). Secondary mitral regurgitation in heart failure: pathophysiology, prognosis, and therapeutic considerations. *Journal of the American College of Cardiology*.

[3] Cioffi G., Tarantini L., De Feo S. (2005). Functional mitral regurgitation predicts 1-year mortality in elderly patients with systolic chronic heart failure. *European Journal of Heart Failure*.

[4] Levine R. A., Hung J., Otsuji Y. (2002). Mechanistic insights into functional mitral regurgitation. *Current Cardiology Reports*.

[5] Montant P., Chenot F., Robert A. (2009). Long-term survival in asymptomatic patients with severe degenerative mitral regurgitation: a propensity score-based comparison between an early surgical strategy and a conservative treatment approach. *The Journal of Thoracic and Cardiovascular Surgery*.

[6] Patel J. B., Borgeson D. D., Barnes M. E., Rihal C. S., Daly R. C., Redfield M. M. (2004). Mitral regurgitation in patients with advanced systolic heart failure. *Journal of Cardiac Failure*.

[7] Trichon B. H., Felker G., Shaw L. K., Cabell C. H., O’Connor C. M. (2003). Relation of frequency and severity of mitral regurgitation to survival among patients with left ventricular systolic dysfunction and heart failure. *The American Journal of Cardiology*.

[8] Fraldi M., Spadaccio C., Mihos C. G., Nappi F. (2017). Analysing the reasons of failure of surgical mitral repair approaches-do we need to better think in biomechanics?. *Journal of Thoracic Disease*.

[9] Nappi F., Avatar Singh S. S., Santana O., Mihos C. G. (2018). Functional mitral regurgitation: an overview for surgical management framework. *Journal of Thoracic Disease*.

[10] Nishimura R. A., Vahanian A., Eleid M. F., Mack M. J. (2016). Mitral valve disease--current management and future challenges. *The Lancet*.

[11] Feldman T., Foster E., Glower D. G. (2012). Percutaneous repair or surgery for mitral regurgitation. *Survey of Anesthesiology*.

[12] Whitlow P. L., Feldman T., Pedersen W. R. (2012). Acute and 12-month results with catheter-based mitral valve leaflet repair: the EVEREST II (endovascular valve edge-to-edge repair) high risk study. *Journal of the American College of Cardiology*.

[13] Stone G. W., Lindenfeld J., Abraham W. T. (2018). Transcatheter mitral-valve repair in patients with heart failure. *New England Journal of Medicine*.

[14] Fanaroff A. C., Zakroysky P., Dai D. (2017). Outcomes of PCI in relation to procedural characteristics and operator volumes in the United States. *Journal of the American College of Cardiology*.

[15] McGrath P. D., Wennberg D. E., Dickens J. D. (2000). Relation between operator and hospital volume and outcomes following percutaneous coronary interventions in the era of the coronary stent. *JAMA*.

[16] McGrath P. D., Wennberg D. E., Malenka D. J. (1998). Operator volume and outcomes in 12,998 percutaneous coronary interventions. Northern new england cardiovascular disease study group. *Journal of the American College of Cardiology*.

[17] CMS (2023). Medicare physician and other practitioners. https://www.cms.gov/Research-Statistics-Data-and-Systems/Statistics-Trends-and-Reports/Medicare-Provider-Charge-Data/Physician-and-Other-Supplier.

[18] Nkomo V. T., Gardin J. M., Skelton T. N., Gottdiener J. S., Scott C. G., Enriquez-Sarano M. (2006). Burden of valvular heart diseases: a population-based study. *The Lancet*.

[19] Enriquez-Sarano M., Akins C. W., Vahanian A. (2009). Mitral regurgitation. *The Lancet*.

[20] Cubeddu R. J., Inglessis I., Palacios I. F. (2009). Structural heart disease interventions: an emerging discipline in cardiovascular medicine. *Journal of Invasive Cardiology*.

[21] Tabata N., Sinning J. M., Kaikita K., Tsujita K., Nickenig G., Werner N. (2019). Current status and future perspective of structural heart disease intervention. *Journal of Cardiology*.

[22] Simpson T. F., Kheiri B., Chadderdon S. (2022). TAVR operator volumes, trends, and geographic variations amongst Medicare beneficiaries in the United States. *Catheterization and Cardiovascular Interventions*.

[23] Chhatriwalla A. K., Vemulapalli S., Szerlip M. (2019). Operator experience and outcomes of transcatheter mitral valve repair in the United States. *Journal of the American College of Cardiology*.

[24] Alkhouli M., Alqahtani F., Holmes D. R., Berzingi C. (2019). Racial disparities in the utilization and outcomes of structural heart disease interventions in the United States. *Journal of the American Heart Association*.

[25] Sparrow R. T., Sanjoy S. S., Lindman B. R. (2021). Racial, ethnic and socioeconomic disparities in patients undergoing transcatheter mitral edge-to-edge repair. *International Journal of Cardiology*.

